# ‘I am afraid the news is not good’ – Breaking bad news in the time of COVID: Experiences from a field hospital

**DOI:** 10.4102/phcfm.v16i1.4256

**Published:** 2024-02-23

**Authors:** Charmaine Cunningham, Pat Mayers, Janet Giddy, Magdaleen de Swardt, Peter Hodkinson

**Affiliations:** 1 Department of Family, Community and Emergency Care, Faculty of Health Sciences, University of Cape Town, Cape Town, South Africa; 2 Faculty of Health Sciences, University of Cape Town, Cape Town, South Africa; 3 Médecins Sans Frontières, Cape Town, South Africa

**Keywords:** COVID-19, palliative care, South Africa, communication, attitude to death, terminal care, qualitative research

## Abstract

**Background:**

The COVID-19 Pandemic had profound effects on healthcare systems around the world. In South Africa, field hospitals, such as the Mitchell’s Plain Field Hospital, managed many COVID patients and deaths, largely without family presence. Communicating with families, preparing them for death and breaking bad news was a challenge for all staff.

**Aim:**

This study explores the experiences of healthcare professionals working in a COVID-19 field hospital, specifically around having to break the news of death remotely.

**Setting:**

A150-bed Mitchells Plain Field Hospital (MPFH) in Cape Town.

**Methods:**

A qualitative exploratory design was utilised using a semi-structured interview guide.

**Results:**

Four themes were identified: teamwork, breaking the news of death, communication and lessons learnt. The thread linking the themes was the importance of teamwork, the unpredictability of disease progression in breaking bad news and barriers to effective communication. Key lessons learnt included effective management and leadership. Many families had no access to digital technology and linguo-cultural barriers existed.

**Conclusion:**

We found that in the Mitchell’s Plain Field Hospital, communication challenges were exacerbated by the unpredictability of the illness and the impact of restrictions on families visiting in preparing them for bad news. We identified a need for training using different modalities, the importance of a multidisciplinary team approach and for palliative care guidelines to inform practice.

**Contribution:**

Breaking the news of death to the family is never easy for healthcare workers. This article unpacks some of the experiences in dealing with an extraordinary number of deaths by a newly formed team in the COVID era.

## Introduction

The COVID-19 pandemic has had profound effects on healthcare systems and healthcare workers (HCWs) around the world. Although remote and online interactions have become the norm in many settings, for patients and families it was particularly difficult not to be able to visit sick and dying relatives or to communicate and build relationships with their HCW as recommended for traditional palliative care.^1^

In the Western Cape, as elsewhere in the world, a key strategy for coping with the surge of COVID-19 cases was setting up dedicated field hospitals to provide additional care.^2,3^ Severe lockdown measures were imposed to prevent the spread of COVID-19, including no-visitation policies at health facilities, which impacted relationship building between HCWs and patients’ families.^4^ A substantial proportion of COVID-19 deaths occurred in field hospitals, without family present. Often the family had not seen their relative since admission, may not have realised the gravity of their clinical condition and had not been prepared by HCWs for the impending death.^1,5,6^

Breaking bad news is a complex learnt skill, which requires empathy, the use of appropriate body language and tone.^7^ In South Africa, there is an added linguo-cultural barrier, which may contribute to miscommunication. Having time to build trust, establish rapport and prepare families, facilitates communicating bad news. When a family is unprepared for bad news, delivered telephonically by someone unknown to them, the family may react with disbelief and display hostile and blaming behaviour.^8^

Palliative care training was minimal in the field hospital setting, comprising limited palliative care guidelines, including a script for end-of-life communication. Several other settings have reported attempts to train HCWs to break news over the phone, including through simulation, but the efficacy of this approach has not been clearly demonstrated.^7,8,9^

This study explored the experiences of HCWs working in a COVID-19 field hospital regarding communicating with families and having to break bad news over the phone.

## Methods

### Study design

A qualitative exploratory design was utilised.

### Setting

The 150-bed Mitchells Plain Field Hospital (MPFH) in Cape Town was set up as the second wave escalated, operating from 01 January to 31 March 2021.^3,10,11^ Some 604 patients were admitted, with 85 deaths in the facility (14% mortality rate).^11,12^ Care was provided by a multidisciplinary team of doctors, nurses, physiotherapists, social workers, carers (lay workers with limited medical training) and dieticians. The hospital operated with a flattened hierarchy, and all members of the multidisciplinary team contributed their experiences and ideas about how the field hospital could best operate.

Patients were referred from acute care hospitals from the greater Cape Town metropole. Patients admitted were at either end of the illness spectrum – either not severely ill and likely to recover without much intervention or they were not candidates for active intervention (including critical care admission), having been triaged for palliative care.

### Population and sampling

The study population comprised HCWs who had worked in the MPFH. Purposive and snowball sampling was utilised. Staff were informed about the study and, if interested, further information was provided. Those who participated were requested to inform other potential participants of the study.

### Inclusion and exclusion criteria

Inclusion criteria: All HCWs who had worked directly with patients in the MPFH.

Exclusion criteria: Staff who were unwilling to participate or unavailable at the time of data collection.

### Data collection

We developed a semi-structured interview guide (Appendix 1), in consultation with the senior clinicians involved in the MPFH and piloted this with two participants. No changes were required, and these interviews were included in the data analysis. Interviews were audio-recorded and conducted by three experienced qualitative female researchers, through a mix of online and face-to-face interviews. The interviews took place after the closure of the field hospital and face-to-face interviews were held at a setting proposed by the participants. None of the interviewers had worked directly with patients in MPFH. Data collection took place between March and April 2021 and were terminated when data saturation had been reached. Interviews were terminated after 11 interviews, with early analysis suggesting there were no new themes emerging in the last interviews, as well as the fact that interviews more than 2 months after the event might be testing recall.^13^

The study adhered to the criteria for reporting qualitative research (COREQ) guidelines for qualitative studies.^14^

### Data analysis

Interviews were transcribed verbatim using Otter.ai (https://otter.ai), and interviewers checked their own interviews for accuracy. Thematic data analysis was conducted using an inductive approach according to Braun and Clarke (2012).^15^ The steps were: familiarising with data and taking notes, generating initial codes, searching for themes, reviewing the potential themes, defining and naming themes and producing a report. The research team met regularly to discuss the coding and emergent themes.

### Ethical considerations

Approval for the study was obtained from the Health Research Ethics Committee of the University of Cape Town (HREC 504/2020) and permission to conduct the study was granted by the Western Cape Department of Health (WC_202010_021). Informed consent was obtained. Participants were assigned codes, and data were kept confidential through password protected files. Counselling services were available should interview questions cause distress to participants.

### Trustworthiness

All team members have qualitative research experience. An audit trail of methodological decisions regarding data collection and analysis was kept. Investigator triangulation of interview transcripts was done to promote credibility. Developing themes and consensus on the final themes was achieved through regular team discussions and consensus, as well as shared experiences of conducting the interviews, which facilitated reflexivity. Two independent research associates reviewed the data analysis and created the themes. The research associates were not involved in the study or interviews; this was triangulated with the analysis conducted within the research team. This study forms part of a larger consortium, and regular check-ins occurred with this group as well.

## Results

Within 2 months following the closure of the MPFH, 11 HCWs were interviewed. Interviews ranged from 30 to 88 min (median 46) (Table 1).

**TABLE 1:** Socio-demographics of participants.

Interview code	Length(minutes)	Gender	Profession	Years post-graduation
A	30	Male	Doctor	< 3
B	39	Male	Doctor	< 3
C	45	Male	Doctor	< 3
D	27	Male	Doctor	< 3
E	44	Female	Doctor	< 3
F	46	Female	Doctor	> 10
G	88	Female	Social worker	< 3
H	81	Female	Social worker	> 10
I	67	Female	Registered nurse	> 10
J	51	Female	Registered nurse	> 10
K	36	Female	Physiotherapist	> 10

**FIGURE 1:**
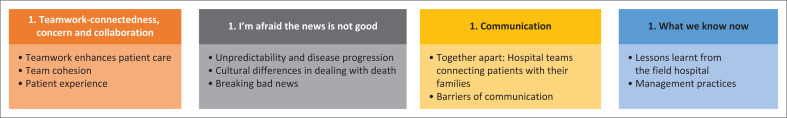
Main themes.

Following research team discussions and a review by an external qualitative research group, four main themes were generated (Figure 1).

### Teamwork: Connectedness, concern and collaboration

#### Teamwork enhances patient care

Teamwork was a prominent theme, with participants sharing that teamwork enhanced patient care and assisted in providing support for the patient and family. This was a new, and uniquely established team, constituted at very short notice and it appeared that how the team collaborated in the MPFH was different from ‘business-as-usual’:

‘I do feel that sort of initially being thrown into a situation where we didn’t know anybody, the staffing, it was all from different areas, or different hospitals being put together. And we sort of had to be a team. And we had to quickly, understand what each other’s roles were, but also having to go through that experience, you know, such a historical thing together, it’s almost like … going to war, you know, and you have your fellow soldiers with you, you don’t really know, the full background, but you fighting this thing together, and you’re in it together.’ (Interview K)

‘… it was just a group, a group of people that were working towards the greatest thing, and it was really was amazingly cool.’ (Interview G)

**Interprofessional teamwork:** Interprofessional collaboration was experienced as being much better than in previous settings where HCWs had worked, especially the way in which people with different roles supported each other:

‘I think, in most cases we would be there to answer, you know, the medical questions, but the nurses and the social worker played a big role, if not a bigger role than we did. Because they were able to provide patients with the sort of support that either we didn’t really know how to, or we weren’t always that good at.’ (Interview B)

Most HCWs had not previously worked closely with social workers and appreciated the key role they played in enabling communication with a patient and their family; this differed from a ‘normal hospital setting’:

‘And when you will communicate as a team, you would have like a ward round where a social worker would come along, and just listen to see if there’s any problems that we’ve identified. And then she would come afterwards and chat to the patients, I think it was important that they were like a very big part of the team. And they would often come to us and tell us about things that we hadn’t noticed or we forgot to ask about. So they would make us aware of things that were important that maybe we were in a rush, we didn’t realize.’ (Interview A)

‘… they [*social workers*] were just such a vital part of the team … It was like we were so busy. And they were always in contact with the families … like WhatsApp with the families … they were also the ones who initiated the video calls with the family. They used to chase family also and so they were really amazing. I loved working with them … they were really good.’ (Interview D)

**Nursing:** In other facilities and circumstances, participants spoke of nurses as being central to breaking the news of death with families, whereas in the MPFH, the nurses were less involved in dealing with the families.

‘the nurses do play a very important role. And a lot of the times they are more with the patients than what the doctors are. So it was nice to sort of share that responsibility because sometimes patients or family do they ask us questions. And we don’t always know the answer because we’re dealing with so many other patients that our time periods very short compared to nursing staff not to say that the nursing staff don’t have lots of patients to deal with too. But a lot of times they can have a better relationship.’ (Interview G)

#### Team cohesion

**Providing emotional support for team members:** Teamwork and collaboration were described as unique, especially in the way that team members coped, participated in debriefing sessions, respected and supported each other:

‘I think that team building, the support, really, really need a team. It’s the respect as well. For each other. As in the multi-disciplinary team. Team building, it’s very important as well. Yeah. And then … our morning meetings, you know, those meetings … they also make that good relationship, you know, you get to know each other.’ (Interview B)

‘We needed each other, because we don’t know what is going to happen to you tomorrow even, or even now. You are here looking after this patient. Things can change. We looked after each other … we have to depend on each other. Yeah we’re all in this together we’re like “I’ve got your back and you’ve got my back.”’ (Interview J)

Dealing with so many deaths was stressful. While each HCW had their own ways of dealing with it, the value of talking to colleagues was highlighted:

‘… everyone had a closer person that they would probably speak to. And I think that worked out so well. So without, without it being intentional. I think everyone was checking up in a roundabout way of everyone.’ (Interview E)

#### Coping with patient anxiety and fear

Participants found that many patients were dealing not only with their own illness and fears but were also worried about their family at home. Sometimes patients were dealing with the death of other family members (and not uncommonly, multiple deaths) and had to cope with their own grieving while being isolated from their support network:

‘A lot of patients, their family members died quite recently. So, they were also dealing with their own grief. And, often, we would only realize a few days later that someone’s husband passed away in the same hospital … so those are just small things that we didn’t think to ask in the beginning. And then it would come to light, like a few days later.’ (Interview A)

‘And it it’s tragic, because this particular family and this is why it resonated with me is because when speaking to the son, he had lost his wife to COVID, he had lost his father to COVID, and I while he was telling me this, I just remember my heart sinking, you know, you know, one individual experiencing so much trauma in such a short period of time.’ (Interview C)

This was aggravated by patients’ sharing a ward with others, all with a COVID-19 diagnosis and observing the condition of some patients deteriorate their ultimate death. The fear of death was constantly present:

‘I think normally, when you work in a hospital environment, not everyone’s got the same diagnosis. So, if someone dies, they might die from something that you don’t have at all. But the tricky part is everyone had COVID. So, when one person died from COVID, you could get that sense that the patients next to them were thinking about ‘were they next’, because they had the same diagnosis. Maybe they don’t understand that, you know, age plays a role, your co-morbidities, but also, we’ve had those incidences where we expect people to die and they live or we expect people to live and they die. So, there was no guarantee that you weren’t next …’ (Interview E)

### Breaking the news of death to family – I am afraid the news is not good

#### Unpredictability and disease progression

Participants expressed frustration that it was difficult to predict the clinical course of COVID-19. Changes in patients’ condition were often rapid and unexpected. The condition of patients prepared for discharge could unpredictably deteriorate and they could die within hours:

‘Quick, quick, quick was changing. So, could you be talking to the patient now and then when you come back “Haibo [*Oh my*] … all of a sudden, this patient now is going down like that …”’ (Interview J)

‘… that made it difficult for us and for the family because you’ve already told the family No, she’s doing well. You’re about to send her home, we just waited for one or this and then suddenly they deteriorate, and they die.’ (Interview F)

**Family members having difficulties dealing with the death of a loved one and blaming staff:** Families reacted in different ways when told over the phone about the death of their loved one. Often their initial reaction was anger and blaming the hospital staff for doing something wrong or not telling them earlier about the gravity of the situation:

‘And then he died. Like so suddenly, right. So, the family was very suspicious, it was a very … the wife, shouted at the doctor and blamed the doctor. So, it was a really, really bad phone call for the doctor.’ (Interview G)

‘I think that that was for me the hardest … to not always be sure what their response is going to be? Always the uncertainty of what the responses is going to be. We always like to know what to anticipate.’ (Interview D)

**Challenges of communicating with families:** Participants sometimes found themselves in the midst of complex family dynamics, with family members not always sharing information among themselves. Families without phones had to be contacted via a neighbour or another family member. This meant that HCWs would often have to repeat information. There was no dedicated person to communicate with the families, and HCWs had to fit these complicated telephone calls into their clinical duties:

‘… you would find that the phone would then be transferred to another person, and then another person and another person. And so, you’re actually breaking the news to numerous people … and I know there were issues numerous times where one family member would perceive some information differently to another family member.’ (Interview G)

#### Cultural differences in dealing with death

Most participants, when asked about cultural differences in the way families responded to bad news, thought there was little difference between cultures (and language groups), with the only comment being that Muslim families may be calm and accepting about death, while other cultures may be more emotional.

#### Breaking bad news

**Preparing the family and building a relationship:** Participants were often conflicted over what to tell the family, because they were unsure about the disease progression and prognosis. Concern about creating false hope for the family made them very careful with their words:

‘Like I said, with those traumatic deaths where you just don’t know at times you think you didn’t handle it the way that you were supposed to but you know, after a day or two, then the family settles down and then they’ll come back to you and give you their most beautiful call where they like, it’s okay, you know, we accepted that you did, everything the hospital did everything I’m so grateful for every day for keeping us in touch.’ (Interview G)

They all agreed that good communication, and time to prepare the family, developed trust and made breaking bad news easier:

‘… we had had such a good communication for the week prior, it was easier to communicate this than if we just phoned her out of the blue … if there wasn’t such good communication, it would, like really just exacerbated grief for the patient and the patient’s family.’ (Interview A)

When there was not enough time to prepare the family, the participants felt guilt as well as grief:

‘I felt like maybe I could have prepared the family more …’ (Interview G)

‘the death was a shock to me. It wasn’t something … that wasn’t something I’d expected. And I also felt sad about the death.’ (Interview B)

**The importance of family members’ visiting the patient:** As the pandemic evolved, the MPFH (along with many other facilities) allowed families to visit patients who were thought to be dying. Despite the protocols and personal precautions required, this was appreciated by everyone, giving patients peace of mind, and families some closure:

‘… when they come and see the family in person, they have a much better understanding of what you were trying to communicate on the phone, and then it really helps with communication, and then onwards.’ (Interview A)

‘… I feel as if family members really need to see how, how their family members are doing at hospital, to sort of grasp the severity.’ (Interview B)

‘And definitely, try to get the families to come visit when the patient is passing away, that definitely helped.’ (Interview F)

### Communication

It was often a challenge for patients to communicate with their families. Only a minority had their own phones with data and were able to communicate regularly. Many were dependent on staff to help them connect and keep their families informed about their condition. Initially audio calls were used, but later video calling was used to connect patients and families.

#### Together apart: Hospital teams connecting patients with their families

**Digital communication to keep families updated:** Phone calls did not allow optimal communication with families when dealing with difficult updates; it was not always possible to know whether the family understood the news:

‘There wasn’t always time. We tried most of the time to give them updates daily, but there were some patients that slipped through the cracks. So, I think for me the biggest challenge, the biggest obstacle for us, was communication with the family. Just to prepare them maybe and tell them about the prognosis because they couldn’t always come.’ (Interview D)

Non-verbal cues and responses were missing in the interaction, and participants were often unsure as to whether the family really understood the severity of the patient’s condition or the bad news:

‘… we just spoke on the phone, it’s very difficult, it’s very difficult, because I think human nature is that we communicate both verbally and non-verbally.’ (Interview C)

‘it’s definitely difficult because you don’t, you don’t have the rapport that you normally have when you’ve got the family in front of you. You can’t see their reactions; they can’t see yours. You don’t know how they are perceiving what you’re saying, if they understand. The phone lines weren’t always that great, either. So, it’s really difficult.’ (Interview F)

**Video calls keeping families connected:** Using a hospital device enabled video calls between patients and their families, which was greatly appreciated:

‘… it can change their entire day, like they would have like been lying in bed all day, but then they just get a video call and they like, woke up … I think it can really change their outlook … it made a very big, and important difference to do the video calls.’ (Interview A)

‘I think that’s one thing that I loved doing … and sitting there doing the video calls and because the video calls allowed the family to be inside when they could not. Yeah, so it was just the most beautiful experience. Suddenly they [*patient*] see all of the loved ones on this phone, and they’ll cry, it will be the most beautiful, beautiful moment.’ (Interview G)

#### Barriers to communication

Communication challenges with families were a key issue for all HCWs. Language barriers, a constant challenge in the South African health system, were aggravated during telephone calls. This made communicating bad news more challenging:

‘… if there’s a language barrier, you can’t have somebody that can translate for you at the same time [*on the phone*]. So, I found it very, very difficult.’ (Interview F)

Despite fluency in a language, communication challenges related to language were also experienced:

‘So even though I am Xhosa, I did my medical education in English. So, I found it to very difficult to explain medically in my home language. So, it was a lot easier in English because I know the terms in English. Yeah … Because obviously, when you speak to a family, you know, you don’t want to use medical terms, but it is very difficult to break it down easily in my home language. Yeah. So, I found that to be particularly challenging.’ (Interview B)

It was sometimes extremely difficult to contact a family:

‘They try to call his wife and then his wife … didn’t pick up the phone. And then the social worker also tried and the doctor also tried but you know when they … when she’d come back? And then he was already gone. So that family – they were very angry.’ (Interview J)

The result of the communication challenges was summed up by one participant:

‘I think in the cases where we didn’t have good communication, we had very upset families.’ (Interview A)

### What we know now: Lessons learnt from the field hospital

#### Teamwork and communication

Team building and communication emerged as some of the key foundations to this setting:

‘Getting the teamwork going is like … before the hospital open, have like a team building session, get to know … so that the team members can just get to know each other a bit quicker, learn each other’s names, and … already decide how they want to run the hospital or the ward … how they want to do things before the time already. That will just help the team work better together, quicker … Getting the communication channels between the social workers and nurses, and the doctors open, and with the family, is I think one of the most important things dealing with death.’ (Interview F)

While communication barriers were frustrating, regular, early updates made a significant difference for participants and families:

‘Just spending time and just putting aside time with those family members who are upset or angry or hostile. I think if we just acknowledge how they feel, and just answer how they feel and not retaliate back with that kind of energy, it makes the world’s difference in avoiding.’ (Interview C)

‘From the very beginning, from the day that the patient arrives in the field hospital from where they come from, I think getting the family updated from that moment is crucial. But in my experience, also, also sharing the fact that COVID is a very unpredictable disease, you know, from the outset.’ (Interview D)

‘Phone the families regularly, have a video call if they want to, and can, so that they can also see if somebody is really sick on admission. And … Ja [*yes*], I think the most important thing is just to contact the families regularly, for updates.’ (Interview H)

#### Need for staff training in providing palliative care

Participants generally felt unprepared for dealing with dying patients, but especially in dealing with their families and breaking the news over the phone:

‘We never had like a formal training on how to [*break bad news*] over the phone.’ (Interview C)

There was an expressed need for further training in palliative care:

‘“Not for escalation” doesn’t mean that they should be left to die, it should just mean that they should be comforted in the remaining days … I don’t think people have been really trained enough to know how to deal with those situations.’ (Interview B)

Support staff also felt the strain of the multiple deaths. This participant felt that support and training should be offered to all hospital workers:

‘Because when come to death it’s affecting everyone. Even the cleaners were cracking, you know, because they were cleaning and talking to this person and all of a sudden, the patient just went down just like that, so it was not affecting only us nurses, like it was affecting everyone. Yeah. And if you are working in a hospital, I think you should go for that training.’ (Interview J)

One participant expressed doubt about the value of formal training and implied that it was something one learns through experience:

‘I don’t think teaching someone like giving a lecture about breaking bad news is ever really going to prepare you for doing it.’ (Interview A)

#### Management practices and senior personnel

Participants appreciated strong and accessible leadership, clear guidelines and standard operating procedures (SOP). The presence and availability of senior clinicians to assist in decision making were valued:

‘We had a very good senior staff as well … people that have experienced death a lot more than you have and can kind of give you advice or just listen to give advice also be important.’ (Interview A)

‘There was always someone to ask for advice. You could always go speak about a bad experience. always speak to them about it. So, I felt very supported by the management and by my seniors.’ (Interview D)

An SOP was developed to assist staff regarding how to talk to relatives about the death of a patient and make the breaking of bad news easier:

‘And what’s also nice is that like, an SOP written out specially if it’s like, one or two in the morning … of what you say exactly. So that was very nice of them to write exactly like … a script About exactly what to say when you’re breaking the bad news. So, I think that that also helped me a lot.’ (Interview D)

## Discussion

### Main findings of the study

Healthcare workers are taught that when the condition of a patient suddenly deteriorates or the patient dies, the family must be contacted and asked to come to the hospital. Only when seated in a safe space, should they be informed that their loved one is likely to die or has died. COVID-19 broke all these dogmas, presenting a challenge to HCWs faced with breaking bad news.^1^

The importance of the team, and how it functioned, emerged as the core thread weaving all the MPFH experiences together. The team was vital whether dealing with work, breaking bad news or coping with emotional distress, which aligns with reports from other COVID field hospitals.^16,17,18^

The leadership of the MPFH was very intent on creating a well-functioning team. Teamwork was experienced in an egalitarian way, without an institutional history and the traditional strong vertical hierarchies.^19^ There were interdependencies between roles that traditionally do not exist. For example, social workers stepped in and conducted difficult conversations if doctors were struggling.^16^ We found an apparently reduced role of nurses in communicating with families, likely because of workload, COVID fears and some role reversal in this setting.^20,21^ This is different from what Felix et al. reported from Brazil that the ‘usual role of the medical team in disclosing difficult news’ changed during COVID such that nurses were pushed into this role.^7^

COVID-19 saw health facilities worldwide dealing with patients’ suddenly facing an unpredictable illness, isolated in hospital and unable to see their family even when facing death.^22^ The condition of patients could deteriorate and they could die suddenly without prolonged illness or warning, complicating discussions about prognosis, timelines and preparation for death.^23^ It is reasonable to ask whether refusing to allow families to visit very ill patients was a rational or reasonable response by hospitals, given that PPE was available, and HCWs themselves were spending many hours in COVID facilities. Was this not an example of extreme medical paternalism (‘We know what is best and we are going to tell you what to do because we are in control’). Towards the end of the time of the MPFH, staff were making plans for relatives to be able to visit, but it was probably a case of ‘too little, too late’. This should be a serious consideration in future situations of this kind.

In the MPFH, with many patients’ being too sick to manage their own communications or lacking access to phones or data to communicate with families, HCWs became the link between patients and families. The success of video-conferencing depended largely on family members’ possessing smart phones with video capacity and airtime or data (which cannot be assumed in resource constrained settings).^24^

Most participants did not perceive major differences in how different cultures reacted to the breaking of the bad news. Although anecdotally HCWs may experience differences in dealing with death in different cultures, this was not an issue in communicating by phone in this setting; this was a potential avenue for future research.^25^ This could be attributed to widespread societal awareness of the mortality risk associated with COVID-19 and therefore possibly normalising the idea that people admitted to a hospital might die.

Respect for the dignity and human rights of the patient encompasses the right to information in a language that the patient (and their family) understands.^26^ This is challenging in South Africa, with 11 official languages, and language can therefore present a barrier to communication. The linguo-cultural barrier was mentioned by some of the HCWs interviewed.^27^

For HCWs, providing a link between patients and family increased their already heavy workload.^22^ Harris et al. described the workload for HCWs during COVID-19 as relentless and never-ending, and similar to our participants they faced limited opportunities to de-stress outside of work because of lockdown restrictions and social isolation.^28^ This lack of ‘breathing space’ may lead to HCWs’ struggling with breaking bad news, compounding the psychological impact of working on the COVID-19 frontline and result in HCWs’ emotionally disengaging and/or feeling overwhelmed by feelings of guilt, sadness and anger.^7,29^ It is also possible that the reduced ability to connect with the external world contributed to how favourably teamwork and team support was experienced by the participants.

### What this study adds

Dealing with multiple deaths over a short period had repercussions on individuals, families, the health system and society, highlighting the need to rapidly integrate palliative care approaches into the health system.^30,31^ The lessons learned about positive teamwork and multidisciplinarity reported here and from other COVID-19 field hospitals can inform better ways of working together in traditional health settings.^19^ Early, transparent communication with families, and understanding that they suffer psychological distress when receiving incomplete information is key.^5^ Under the circumstances of the pandemic (and an everyday constraint in low resource healthcare settings), there were not enough resources to schedule daily communications with all families. This was not a task that could be delegated to volunteers or lay-carers.

Although breaking bad news is now an integral part of most healthcare professional training, it is necessary to teach how to do it using different methods, including remotely.^8,9^ Healthcare providers planning for future pandemics can learn not only from the dynamic multidisciplinary team but also mainstreaming regular, high-quality communication with families.

Breaking the news of death to families is not unique to an identified ‘palliative patient’ but is equally relevant to dealing with unexpected deaths throughout the healthcare system. This function is often performed by a junior, inexperienced person, unknown to the family. The importance of how such devastating news is broken to families, and their consequent perceptions of the quality of healthcare, as well as their own bereavement, is clear from local research.^32^

### Strengths and weaknesses and/or limitations of the study

This is the first article describing breaking bad news remotely in a field hospital setting from South Africa. The study relied on snowball sampling of HCWs who provided care at the MPFH. There was a possibility of bias in the sample of those agreeing to participate although this is unlikely given that those we were able to contact not only willingly discussed their experiences but expressed that it was positive.

By the time we completed interviews and started analysis, the field hospital had closed making it difficult to reengage with participants, many of whom were by then employed elsewhere. So conventional member checking was not undertaken, although the value of this is in question, and we believe there was adequate rigour given the insights of the research team. Participants included only two nurses. Despite actively trying to recruit nurses, many had moved to a new employment, were not contactable or declined to participate. In the MPFH, calls to families were largely made by other HCWs, whose experiences were explored.

Finally, the research team consisted of predominantly female doctors and nurses, who may have introduced their own positionality to the analysis. We attempted to mitigate this through the external review by two social scientists from another province.

## Conclusion

Communication challenges in breaking bad news were intensified by the unpredictability of COVID-19, the large number of deaths and the impact of restrictions on families visiting. The importance of the team, transparent communication and the value of strong leadership and intentional actions to build a well-functioning team was highlighted.

## Appendix 1

### Interview schedule

1. Introduce yourself, where you have previously worked, experience, how did you come to work at the hospital.
2. What is your experience in dealing with death and dying before working here?
3. The MPFH dealt with many deaths in the over the last few months. Can you talk about your experiences in dealing with COVID patients in the days around their death, and dealing with their families?
4. Please tell me about an experience that you had during your time at the hospital which really resonated with you as you reflect on this?
5. Can we talk more about when you felt positive about the way a death was handled?
6. How did you yourself feel during and after these episodes?
7. How did you cope with the experiences *Probe – talking to someone, colleagues, counsellors etc*
8. Most health professionals have been trained to avoid breaking bad news over the phone where possible and only to do it in person. But COVID has changed this practice radically. Do you feel your past experiences and training prepared you for breaking news over the phone? Can you tell me about an experience in which you had to break bad news over the phone.
9. Can you contrast the experience of sudden and unexpected deaths vs expected deaths?
10. Video-calling devices were available and used in some cases at MPFH – how did you feel about these? *Probe – helpful? When? Who? How?*
11. In some cases, family members were allowed to visit an obviously palliative patient to “say their goodbyes”. How was this for you? Dealing with patient, family, staff
12. In your capacity as (doctor/nurse/SW etc) how did you experience the roles of the other staff members with whom you worked *Probe: tell me about an experience of working with another professional colleague in dealing with death and dying*
13. What do you feel the key learning points from your experiences are that we can take forward to the next wave/pandemic/everyday practice?
14. In reflecting on the experience – are there aspects that you think the hospital and staff could have changed or done differently? *Probes: - w.r.t patient care; connecting with families, (can add more here)*
15. Anything else you would like to add?
