# Erratum: ‘I am afraid the news is not good’ – Breaking bad news in the time of COVID: Experiences from a field hospital

**DOI:** 10.4509/phcfm.v16i1.4509

**Published:** 2024-04-10

**Authors:** Charmaine Cunningham, Pat Mayers, Janet Giddy, Magdaleen de Swardt, Peter Hodkinson

**Affiliations:** 1 Department of Family, Community and Emergency Care, Faculty of Health Sciences, University of Cape Town, Cape Town, South Africa; 2 Faculty of Health Sciences, University of Cape Town, Cape Town, South Africa; 3 School of Nursing, Faculty of Community and Health Sciences, University of the Western Cape, Cape Town, South Africa; 4 Médecins Sans Frontières, Cape Town, South Africa

In the published article, ‘I am afraid the news is not good’ – Breaking bad news in the time of COVID: Experiences from a field hospital. *Afr J Prm Health Care Fam Med.* 2024;16(1):a4256. https://doi.org/10.4256/phcfm.v16i1.4256, there was an error regarding the affiliation(s) for Pat Mayers.

As well as having affiliation Faculty of Health Sciences, University of Cape Town, Cape Town, the author should also have, School of Nursing, Faculty of Community and Health Sciences, University of the Western Cape, Cape Town.

The publisher apologises for this error. The correction does not change the study’s findings of significance or overall interpretation of the study’s results or the scientific conclusions of the article in any way.

